# Supplementary material for: Origin identification of migratory pests (European Starling) using geochemical fingerprinting
Source: PeerJ. 2020 May 4;8:e8962. doi: 10.7717/peerj.8962 (PMC7204882; doi:10.7717/peerj.8962)
Supplement: Table S1 [file peerj-08-8962-s001.docx]

| **Sampling sites** | **Code** | **Geologic eras** | **Underlying Geology** | **Group** | **Reference** |
| --- | --- | --- | --- | --- | --- |
| Salmon Arm | Md | Cenozoic/pre-fraser glaciation | **Morainal deposits:** till with minor and sand, gravel and silt, drumlinoid (Characterized by streamlined forms) |  | Fulton et al, 1974 |
| Mara | UTsp | Mesozoic/ Upper Triassic | Phyllite, argillite, quartzite; grey to balck; minor tuffaceous rocks | Slocan group | Okulitch, 2013 |
| Hullcar | UTNv | Mesozoic /Upper triassic | Breccia, tuff, flows, augite porphyriy | Nicola group | Okulitch, 2013 |
|  | uTSc | Mesozoic/Upper triassic | Limestone, calcareous, fine-crystalline, siltstone, calcareouos; shale interbeds | Slocan group | Okulitch, 2013 |
| Armstrong | UTsp | Mesozoic/ Upper Triassic | Phyllite, argillite, quartzite; grey to balck; minor tuffaceous rocks | Slocan group | Okulitch, 2013 |
| Vernon | MJmzd | Mesozoic/Middle Jurassic | **Wood lake pluton:** monzonite, biotite and/or hornblende, massive to weakly foliated, medium to coarse grained, equigranular, in part megacrystic; quartz-monzonite; diorite; quartz-diorite; granodiorite; granite |  | 166.4-158.8Ma, Glombic, 2005; Okulitch, 2013 |
|  | Ey | Cenozoic/Paleogene eocene | **Coryell syenite:** syenite, alkalic to calc-alkalic, pink and buff, quartz monzonite; porphyry, trachyte, feldspar, dykes; epizonal; feeder dykes to Marron Formation, Yellow Lake and Kitley Lake members |  | Ca.53-48Ma, Church, 2002; Glombick, 2005; Okulitch, 2013 |
| Kelowna | Eva | Cenozoic/Paleogene eocene | Lava, andesite, with quartz-filled amygdales: breccia, brown | Yellow Lake Member | Okulitch, 2013 |
|  | EWLv | Cenozoic/Paleogene eocene | Breccia, rhyolite; pyroclastic rocks; lahars; minor lava, trachyte and andesite; possibly associated with Marama Formation domes; in type locality: conglomerate, volcanic; sandstone; shale; lacustraine and subaerial | Angular unconformity/ white lake formation | Okulitch, 2013 |
| Penticton | EJgd | Mesozoic/Early Jurassic | **Bromley Batholith:** Granodiorite, hornblende, biotite, marginal diorite; quartz gabbro; garnet skarn  Pennask Batholith: granodiorite, biotite |  | Parrish and Monger 1992; Okulitch, 2013 |
|  | Ggn | Proterozoic to Cenozoic | **Okanagan gneiss:** orthogneiss, granodiorite, hornblende-biotite; grades to gneiss, mylonitic; mylonite; blastomylonite; minor gabbro, amphibolite, paragneiss |  | Okulitch, 2013 |
|  | ESC | Cenozoic/Paleogene eocene | Shingle creek porphyry: intrusive porphyry, quartz; Includes Coryell subvolcanic rhyolite porphyry |  | 52.4 Ma, Church, 2002; Glombick et al. 2004 |
| Keremeos | CTKs | Paleozoic and Mesozoic | Quartize, massive; quartzite, micaceous, foliated; chert, recrystallized; limestone, crystalline; phyllite, argillaceous; minor schist, micaceous | Kobau Group | Okulitch, 2013 |
|  | CTKv | Paleozoic and Mesozoic | Phyllite, chloritic, actinolitic; greenstone; minor serpentinite; marble lenses | Kobau Group | Okulitch, 2013 |
| Oliver | Jqm-b | Mesozoic/Early and/or Middle Jurassic | Quartz monzonite, biotite, minor garnet, hornblende, foliated, syn-kinematic |  | Unit VI of Ryan, 1973 |
| Osoyoos | LTgd | Mesozoic/Triassic | **Osoyoos lake gneiss:** granodiorite, hornblende-biotite, gneissic |  | Ryan, 1973; Parkinson, 1985 |
|  | LJgd-m | Mesozoic/Jurassic | Granodiorite, hornblende, chloritized, sheared, fractured | Osoyoos granodiorite | Part of unit IX, Ryan 1973 |

References:

Church, B.N., 2002 Geology of the Penticton Tertiary Outlier. B.C. Ministry of Energy, Mines and Petroleum Resources, Geoscience Map 2002-05, scale 1:50 000

Glombick, P.M., 2005. Mesozoic to early Tertiary tectonic evolution of the Shuswap metamorphic complex in the Vernon area, southeastern Canadian Cordillera. Unpublished Ph.D. thesis, Department of Earth and Atmospheric Sciences, Universiyt of Alberta (incudes maps, scale 1:50,000).

Glombick, P.M. and Thompson, R.I., 2004. Geology, Creighton Creek, British Columbia. Geological Survey of Canada, Open File 4371, scale 1:50 000

Okulitch, A. V. (2013) Geology, Okanagan Watershed, British Columbia. Geological Survey of Canada.

Parkinson, D.L., 1985. U-Pb geochronology and regional geology of the southern Okanagan Valley, British Columbia: the western boundary of a metamorphic core complex. Unpublished M.Sc. thesis, Department of Geology, University of British Columbia (includes maps, scale 1:25 000)

Parrish, R.R. and Monger, J.W.H., 1992. New U-Pb dates from southwestern British Columbia. In Age and Isotope Studies; Report 5; Geological Survey of Canada, Paper 91-2, pages 87-108.

Ray, B.D., 1973. Structural geology and Rb-Sr geochronology of the Anarchist Mountain area, south-central British Columbia. Unpublished Ph.D. thesis, Department of Geology, University of British Columbia (includes maps, scale 1:31 680).

Surficial geology, Shuswap Lake, west of sixth meridian, British Columbia; Fulton, R J; Berti, A A; Smith, G W. Geological Survey of Canada, "A" Series Map 1391A, 1974, 1 sheet; 1 CD-ROM, <https://doi.org/10.4095/109073> (Open Access)
