# Supplementary figures and images for: Origin identification of migratory pests (European Starling) using geochemical fingerprinting
Source: PeerJ. 2020 May 4;8:e8962. doi: 10.7717/peerj.8962 (PMC7204882; doi:10.7717/peerj.8962)

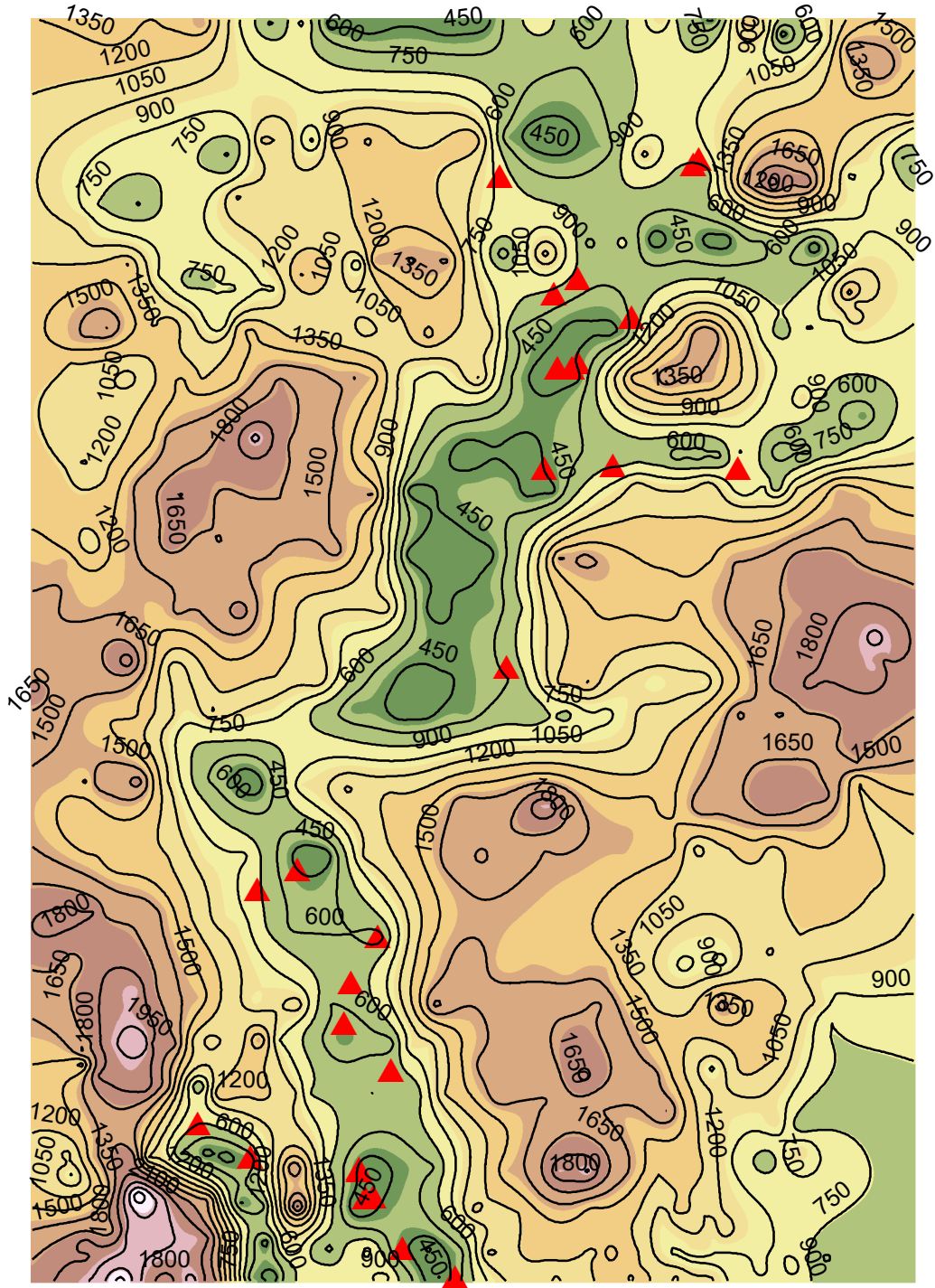

Supplement: Figure S1 [file peerj-08-8962-s002.pdf]

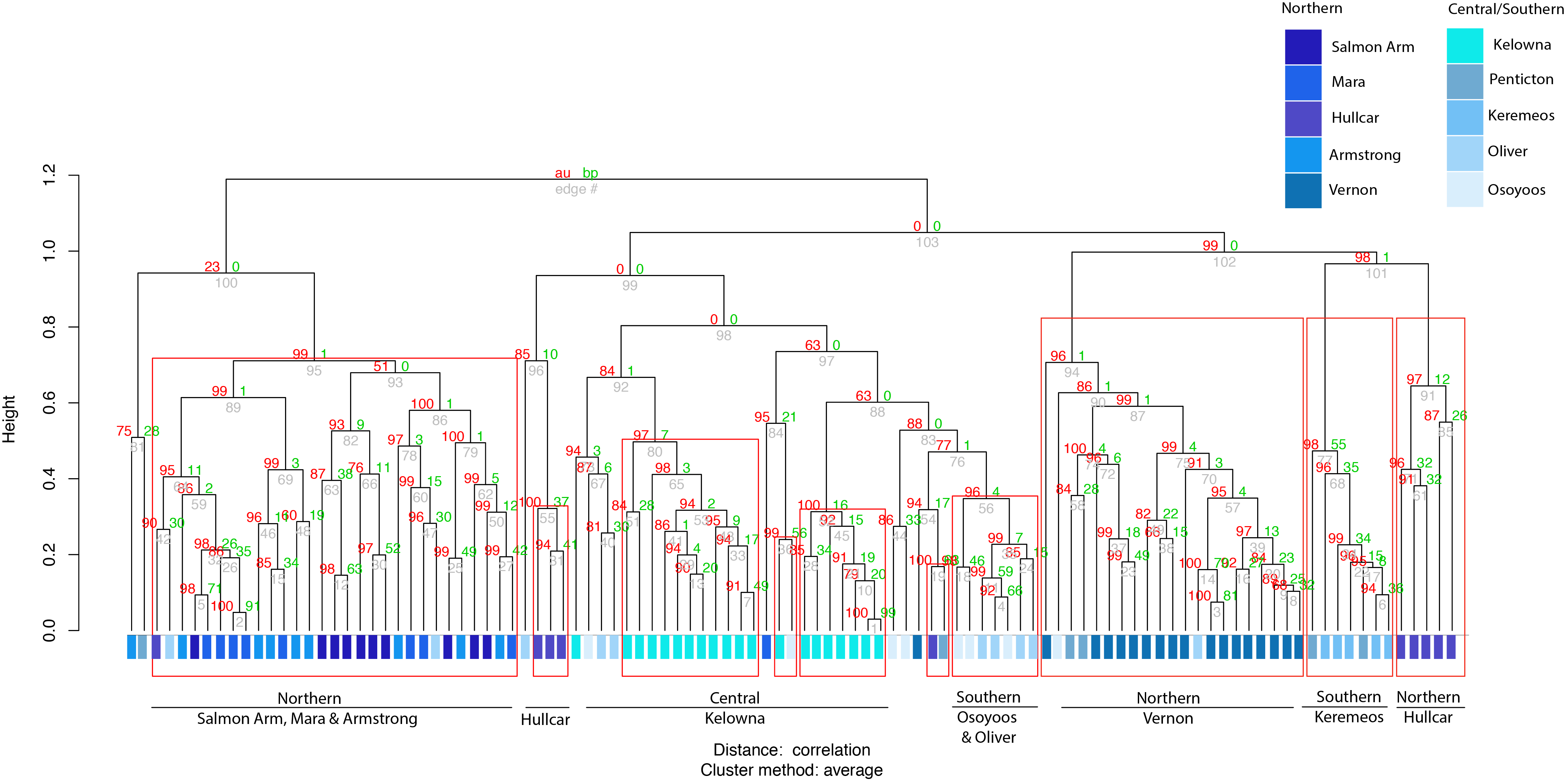

Supplement: Figure S2 — The average method was used to calculate the AU value of 105 juvenile birds sampled in different locations in the Okanagan-Similkameen region, British Columbia in 2015 where the height represents the correlation; same legend as preceding figure; dark blue colour represents samples from the northernmost sites and the colour fades as the location moves towards the south. [file peerj-08-8962-s003.png]
